# Weekly gemcitabine plus Epirubicin as effective chemotherapy for advanced pancreatic cancer: a multicenter phase II study

**DOI:** 10.1038/sj.bjc.6600482

**Published:** 2002-08-27

**Authors:** B Neri, G Cini, L Doni, C Fulignati, M Turrini, D Pantalone, E Mini, C De Luca Cardillo, L M Fioretto, A S Ribecco, R Moretti, M Scatizzi, G Zocchi, A Quattrone

**Affiliations:** Department of Internal Medicine, Oncological Day Hospital, University of Florence, Italy; Department of General Pathology and Experimental Oncology, University of Florence, Italy; Department of General Surgery, University of Florence, Italy; Department of Clinical Pharmacology, University of Florence, Italy; Department of Radiation Oncology, University of Florence, Italy; Clinical Oncology Unit, Santa Maria Annunziata Hospital, A.S.L. 10 Florence, Italy; Department of Surgery, Careggi Hospital, Florence, Italy; Blanchette Rockefeller Neurosciences Institute, Johns Hopkins University, Rockville, Maryland, USA

**Keywords:** chemotherapy, clinical benefit, epirubicin (EPI), gemcitabine (GEM), tumour response

## Abstract

The current role of chemotherapy in pancreatic carcinoma is limited, and progress in the treatment of this disease represents a significant challenge to medical oncology. The most promising drug under study is gemcitabine, a relatively new antimetabolite that represents an attractive candidate for combination chemotherapy because of its excellent side-effect profile and the absence of overlapping toxicities with other chemotherapeutic agents. Combined administration of gemcitabine and anthracyclines could result in the induction of DNA breaks that are not easily repaired by the cell's machinery, thus enhancing the apoptotic signals triggered by these lesions. Forty-four patients with locally advanced and/or metastatic pancreatic adenocarcinoma were enrolled in this multicenter study. Patients received Epirubicin 20 mg m^−2^ for 3 weeks followed by 1 week of rest (1 cycle) and gemcitabine 1000 mg m^−2^ after Epirubicin on the same day. All were assessable for toxicity and response, 11 patients responded to treatment with one complete response and 10 partial responses, for an overall response rate of 25%. Median survival was 10.9 months (range, 2–26 months). Therapy was well tolerated, with a low incidence of haematologic grade >2 toxicity. A total of 12 of 27 (44.4%) eligible patients attained a clinical benefit response. Our findings suggest that the gemcitabine-epirubicin schedule is active and well tolerated in patients with advanced pancreatic cancer.

*British Journal of Cancer* (2002) **87**, 497–501. doi:10.1038/sj.bjc.6600482 www.bjcancer.com

© 2002 Cancer Research UK

## 

Locally advanced and/or metastatic pancreatic cancer is an aggressive and rapidly fatal disease that represents the fifth most common cause of cancer-related mortality in the United States and Europe (Jensen *et al*, 1990; Parker *et al*, 1997). Although advances have been made in the diagnosis and treatment of many gastrointestinal malignancies, no such progress has been made in pancreatic cancer. Consequently, median survival from the time of diagnosis ranges from 3 to 6 months for patients with unresectable disease (Brennan *et al*, 1993).

Treatment with single-agent 5-fluorouracil (5-FU) produces tumour response rates in the range of 0–20%, with little additional response when used in combination therapy, and no impact on survival (Brennan *et al*, 1993). In locally advanced pancreatic cancer, the combination of chemotherapy with radiotherapy has not gained much support (Klaassen *et al*, 1985). These disappointing results underline the need for new active agents in pancreatic cancer. Among these, paclitaxel, docetaxel, topotecan, and temozolomide have shown only limited activity, with response rates of 5–17% in phase II studies (van Riel *et al*, 1999). In contrast, gemcitabine (GEM), a relatively new nucleoside antimetabolite that inhibits DNA synthesis through a number of mechanisms (Heinemann *et al*, 1990; Heinemann *et al*, 1998), appears to have different properties and activity across a broad range of solid tumours. In particular, GEM has been more effective than 5-FU in the alleviation of some disease-related symptoms and prolongation of survival in patients with advanced pancreatic cancer (Burris *et al*, 1997).

On the basis of preclinical studies, GEM represents an attractive candidate for combination chemotherapy because of its excellent side-effect profile and the absence of overlapping toxicities with other chemotherapeutic agents. Moreover, due to its chain termination masking activity, GEM directly inhibits DNA repair (van Moorsel *et al*, 1997), which could represent a molecular basis for synergistic activity with other DNA-damaging chemotherapeutic agents.

Two of the most potent and widely used DNA-damaging anticancer drugs are doxorubicin and its derivative epirubicin (EPI), whose primary mechanism of action, as with other anthracycline derivatives, is the inhibition of topoisomerase II-mediated DNA resealing and the production of stable DNA breakage. *In vitro* studies in solid tumor cell lines have demonstrated that pretreatment of cancer cells with doxorubicin, followed by administration of GEM, inhibits proliferation and increases the rate of DNA fragmentation. Thus, the combined administration of GEM and anthracyclines could result in the induction of DNA breaks, which are not easily repaired by the cell's machinery, thereby enhancing the apoptotic signals triggered by these lesions (Angelucci *et al*, 1997). These data suggest that a treatment schedule of an anthracycline derivative (EPI) in combination with GEM treatment may be of great therapeutic value in pancreatic cancer. Moreover, because advanced-stage pancreatic cancer is frequently accompanied by debilitating symptoms (Schnall and Macdonald, 1996), we designed a phase II trial to quantify the effect of GEM-EPI administration on clinical benefit response as well as objective responses and median survival in patients with advanced pancreatic cancer.

## PATIENTS AND METHODS

### Patient eligibility

Patients eligible for the trial had a histologic or cytologic diagnosis of pancreatic carcinoma with locally advanced unresectable or metastatic bidimensionally measurable disease. Other eligibility criteria included age ⩽75 years; Karnofsky's performance status >40; no prior chemotherapy, hormone therapy or radiation therapy was allowed.

Baseline haematologic requirements included a leukocyte count ⩾3500 μl, hemoglobin ⩾9.5 g dl^−1^, and platelet count ⩾100 000 μl. Patients were required to have a total bilirubin ⩽2.0 mg dl^−1^, aspartate aminotransferase (AST) and alanine aminotransferase (ALT) ⩽3×the respective upper limit of normal (ULN), and prothrombin and activated partial thromboplastin times ⩽1.5×ULN. A serum creatinine ⩽1.5 mg dl^−1^ and a measured serum calcium ⩽11.0 mg dl^−1^ were also required. A history of myocardial infarction, heart failure, angina, arrhythmia, or severe hypertension excluded patients from the study.

The study was performed in compliance with the clinical standards set forth in the Declaration of Helsinki of 1975. Signed and witnessed informed consent was obtained from each patient prior to entering the study.

### Patient characteristics

Between January 1998 and December 1999, a total of 44 patients, with a median age of 59 years, from six different institutions were enrolled in this trial. All patients had a histologic or cytologic diagnosis of pancreatic adenocarcinoma. At study entry, most patients (86%) had a Karnofsky performance score ⩾60, locally advanced disease (55%), and stage IV disease (77%). Patient characteristics are detailed in Table 1

**Table 1:**
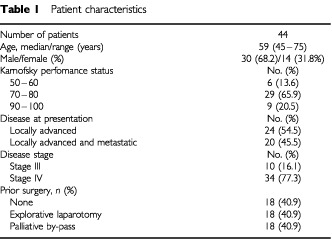
Patient characteristics

.

Forty-four patients were assessed for toxicity and response. Two patients elected to discontinue treatment during the first and second cycles of chemotherapy. Out of the 42 evaluable patients, 27 (64%) with tumour-related symptoms, such as pain, weight loss, and impaired performance status, were also assessable for clinical benefit response.

### Treatment

EPI 20 mg^2^ was administered as an intravenous bolus injection on day 1, 8, 15 for 3 weeks followed by a week of rest (1 cycle); GEM diluted in normal saline solution at the dose of 1000 mg m^−2^ was administered intraveously over 30 min after EPI on the same day. Concomitant medications routinely administered before cytotoxic drugs included ondansetron 8 mg and methyl-prednisolone 40 mg intravenously. Treatment courses continued in patients who achieved objective response, stable disease, or positive clinical benefit response until a total of seven courses or until there was significant clinical deterioration because of tumour-related symptoms. Patients were not allowed to receive concomitant radiation therapy or hormonal therapy during the trial.

The toxicities of each course were recorded before the next course was started and were graded according to World Health Organization (Miller *et al*, 1981) criteria. Chemotherapeutic drug doses were reduced by 25% in the subsequent cycle if the lowest WBC (absolute granulocyte) count was less than 1000 μl (500 μl), the lowest platelet count was less than 50 000 μl, or if any severe (WHO grade ⩾3), nonhematologic toxicity was observed in the previous cycle. Treatment could be delayed for up to 2 weeks if the WBC count was lower than 3000 μl and/or a platelet count less than 75 000 μl. Granulocyte colony-stimulating factor (G-CSF) using a 5-day administration schedule was allowed and if haematological recovery was complete the dose of the following cycle was re-escalated to 100%-dose.

### Baseline and treatment assessments

Before entering the study, all patients underwent a full history and physical evaluation, complete blood count with differential routine blood chemistries and ECG and echocardiography to evaluate cardiac function. Chest X-ray, abdominal ultrasound or computerized tomography (CT) scan were performed to define the measurable lesions before the start of treatment, every two cycles to evaluate response and after the seven cycle. Baseline performance status, analgesic consumption, and pain intensity data were also collected.

Objective tumour assessments and evaluation of clinical benefit response, a composite measure of pain (analgesic consumption and pain intensity), performance status, and body weight (Burris *et al*, 1997), were determined at the end of every 2 cycles during chemotherapy and every 3 months after discontinuation of treatment. Tumour measurements were based on the sum of the products of the bidimensional diameter of the lesions. To be classified as a complete responder (CR), a patient had to have complete regression of the disease and be free of symptoms related to the carcinoma for a minimum of 4 weeks. Patients with greater than a 50% reduction in lesion size and no new lesions were classified as partial responders (PR) and minor response for reduction in lesion size <50%. Patients were rated as having progressive disease (PD) if any new lesion appeared, if tumour size increased by 25% over pretreatment measurements, or for deterioration in clinical status consistent with disease progression. Patients who failed to meet the criteria of CR, PR, or PD, and who remained on-study for at least 2 months, were classified as having stable disease (SD). Two objective measurements that showed a response at least 4-week intervals were required to confirm a patients' response and were assessed by CT and/or abdominal ultrasound examination: all tumour measurements in patients who responded were reviewed and confirmed by an independent radiologist blinded to the sequencing of the scans.

### Clinical benefit response criteria

Clinical benefit assessments included evaluations of pain intensity, analgesic consumption, performance status, and weight (Rothenberg *et al*, 1996; Burris *et al*, 1997). Analgesic consumption was computed on a weekly basis as the mean of the daily analgesic consumption, expressed in terms of morphine equivalent mg day^−1^. To be considered clinical benefit responders, patients had to have more than 1 of the following: a 50% decrease in pain intensity; a 50% decrease in analgesic consumption; or a >20-point increase in performance status that was sustained for more than 4 weeks, without deterioration in any of the other parameters. For patients who were stable in pain intensity, analgesic consumption, and performance status, a 7% increase in dry body weight was required for patients to be classified responders. During the study, pain intensity was measured daily by the patients using a 10-cm linear analog scale, and analgesic consumption was based on a daily diary kept by patients. Performance status and weight were measured weekly by a nurse. Each patient was classified as positive, stable, or negative for each of the primary clinical benefit measures. In all cases, positive indicated a sustained (>4 weeks) improvement over baseline in at least one variable, without a negative result in any other variable.

### Statistical analyses

Using standard statistical methods (Gehan, 1961), we used a 2-stage design in this study. If no CR or PR was noted in the first 14 patients, a response rate of >20% could be excluded with 95% confidence and accrual was stopped. If at least 1 CR or PR was observed, an additional 30 patients were entered in the study for a target sample size of 44 patients. *P*-values were calculated from the student *t*-test and values <0.05 were considered significant. For overall response rate, 95% confidence intervals (CI) were calculated as described by Anderson *et al*, 1982. Time to response was measured from the first dose of chemotherapy to the onset of best response, duration of response was measured from the onset of the best response to the date of disease progression, and overall survival from the first dose of chemotherapy to the date of death.

## RESULTS

### Tumour response and survival

Eleven patients responded to treatment, with 1 (2%) CR that lasted for 26 months without additional therapy and 10 (23%) PR, for an overall response rate of 25% (95% CI, 19–34%). The median time to response was 2.3 months (range, 1.5–4.1 months), and the median duration of response was 11.4 months (range, 13–26 months). An additional 18 (41%) patients reached SD, including two who achieved minor responses lasting for a median of 6.1 months (range, 2.5–14.5 months). The remaining 13 patients (30%) progressed. Out of the 44 enrolled patients, two discontinued treatment after the first and second cycle of the remaining 42, 38 were dead and four were alive as of the data cut-off date. The median time to progression was 4.1 months (range, 1.9–19 months). The median survival duration was 10.9 months (range, 2–26 months), and the probability of surviving beyond 12 months was 23%.

### Clinical benefit response

A total of 12 of the 27 (44.4%) patients with symptomatic pancreatic cancer were classified as clinical benefit responders; nine patients (33.3%) experienced worsening of at least one variable. Eight (29.6%) patients experienced an improvement in performance status that was sustained for at least four weeks during the study period. Nine (33.3%) patients suffering from pain at study entry experienced a reduction of pain intensity and/or analgesic use. With regard to weight gain, six (22.2%) patients had a positive response (>7% increase from baseline). The median time to clinical benefit response was 5 weeks (range, 3–10 weeks), and the median duration was 28 weeks (range, 6–76 weeks).

Out of the 11 patients who achieved objective tumour responses, only six were symptomatic, and of these, five were clinical benefit responders. We did not observe a correlation between clinical benefit response and tumour response (*P*=n.s). However, for the patients who achieved a clinical benefit response, median survival was 9.6 months compared to 6.5 months for clinical benefit non-responders. While there were no differences in terms of response rate, survival and clinical benefit response between patients with locally advanced *vs* metastatic disease.

### Dose administration and toxicity

Patients received a total of 263 chemotherapy cycles. Thirty (71%) patients received all of the planned seven cycles of GEM-EPI, while the remaining patients received a median of four cycles (range, 2–6 cycles). The mean total dose of drugs administered was 320 mg m^−2^ (range, 120–420 mg m^−2^) for EPI and 12 000 mg m^−2^ (range, 6000–21 000 mg m^−2^) for GEM. A total of 85% of dose administrations were given on schedule, 6% were delayed, 5% were reduced and 4% omitted.

The maximum WHO grades (grades 3) encountered during any cycle of therapy are summarized in Table 2

**Table 2:**
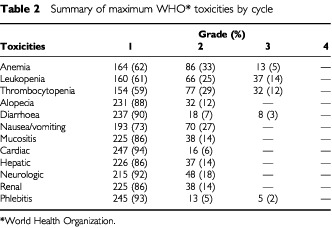
Summary of maximum WHO* toxicities by cycle

. With regard to bone marrow toxicity, the treatment led to a suppression of leukocytes and platelets (14 and 12% of cycles affected, respectively), as well as a significant decline in hemoglobin (anaemia in 4.8% of cycles) and only one patient required RBC transfusion. Myelosuppression tended to be cumulative, with lower and more prolonged nadirs after four cycles. None of the patients required hospitalization for sepsis referable to biliary tract infections or neutropenic fever, and the six who experienced infections (mainly pulmonary) were all manageable on an outpatient basis.

Only three patients had grade 3 gastrointestinal toxicity (diarrhoea). Mild nausea/vomiting (grade 1/2) occurred in 20 patients (27% of cycles), and only a few patients had minor hair loss (grade 1/2 in 20 patients and 12% of cycles). Renal, hepatic and neurologic toxicities were generally mild, only one patient had grade 3 chemically-induced phlebitis. The mean total dose of EPI administered was 320 mg^2^ and no evidence of cardiac toxicity (WHO>2) was recorded.

## DISCUSSION

Pancreatic adenocarcinoma is generally considered resistant to cytotoxic therapy. As with other gastrointestinal malignancies, 5-FU is the most widely investigated agent and produces tumour response rates ranging from 0–20% and median survival times of 4.2–5.5 months (Brennan *et al*, 1993). Modulation by folinic acid, protracted venous infusion, or timing with circadian rhythms seem to have only marginal effects on response rates and survival. Recent phase II studies have reported promising clinical activity with the combination of GEM and anthracycline derivatives in advanced breast and non-small cell lung cancer (Lueftner *et al*, 1998; van Putten *et al*, 2000). In a phase II trial by Scheithauer *et al*, 1999, 70 patients with metastatic pancreatic adenocarcinoma received 4 weekly courses of GEM 1000 mg m^−2^ on days 1, 8, 15 plus EPI 60 mg m^−2^ on day 1. Out of the 66 patients evaluable for response, there was an overall objective response rate of 21% (1 CR, 13 PR). Median survival was 7.8 months. We therefore decided to combine GEM and EPI, as proposed by Scheithauer, but with a different schedule. In our trial, patients received a weekly administration of GEM plus EPI once weekly for 3 consecutive weeks in an attempt to exploit the synergistic action of the two drugs while minimizing the overall toxicity. This regimen was associated with promising activity in our patients with advanced pancreatic cancer. Twenty-five per cent of patients attained an objective response, and median survival for responding and nonresponding patients combined was 10.9 months. Recently, there has been renewed interest in single–agent chemotherapy with the advent of novel agents such as irinotecan (response rate 11%) (Sakata *et al*, 1993) and docetaxel (response rate 20%) (Androulakis *et al*, 1999). Single-agent GEM has also been recently investigated for activity as well as toxicity in advanced pancreatic cancer by Casper *et al*, 1994 and Carmichael *et al*, 1996, who reported modest objective response rates of 6.3–11% and median survivals of 5.6–6.3 months; however, the symptomatic improvements were greater than the objective tumour response rates would suggest. Consequently, the National Cancer Institute and the Food and Drug Administration (USA) have accepted that the relief of tumour-related symptoms is itself a noteworthy goal of carcinoma treatment (O'Shaughnessy *et al*, 1991). Given the palliative nature of advanced disease, we reasoned that pancreatic carcinoma offered an appropriate clinical setting in which to examine these alternative endpoints. Thus, the current study evaluated clinical benefit response as an endpoint of efficacy. We observed a palliative effect in 44% of symptomatic patients; a similar rate (43%) was reported by Scheithauer *et al*, 1999. Yet, consistent with Hidalgo *et al*, 1999; half of patients who attained clinical benefit did not achieve an objective tumour response.

The results of this study also clearly demonstrated that the GEM-EPI regimen was adequately tolerated, and was associated with moderate toxic effects that were qualitatively and quantitatively similar to those reported in other malignancies (Lueftner *et al*, 1998; van Putten *et al*, 2000). Leukopenia WHO grade 3 was observed in 14% of patients; however, there were no episodes of febrile neutropenia requiring hospitalization. Other common nonhaematological toxicities such as nausea/vomiting and alopecia were mild, and we did not observe any cardiac toxicity at this dose of EPI.

In conclusion, this study demonstrates that weekly GEM plus EPI is well tolerated and confirms the observation of symptomatic improvement in a reasonable proportion of patients with advanced disease. In addition, the regimen is active with a good response rate, and a long median survival duration. Our future efforts will focus on randomized trials evaluating this GEM-EPI schedule in patients with earlier-stage disease and/or as adjuvant chemotherapy.
